# Can heat conditions affect the heart rate responses, perception of effort, and technical performance of young male football players during small-sided games? a comparative study

**DOI:** 10.1186/s13102-024-00970-x

**Published:** 2024-08-19

**Authors:** ZhiHui Kang, Zhongju Chen, GuiYang Liu

**Affiliations:** 1 Public Sports Department, Ningbo University of Finance and Economics, Ningbo, 315175 Zhejiang China; 2 School of Physical Education, Chizhou University, Chizhou, 247000 Anhui China; 3 Physical Education and Health Education, Udon Thani Rajabhat University, 64 Thaharn Road, Muang, Udon Thani, 41000 Thailand

**Keywords:** Soccer, Sided games, Hot temperature, Body temperature regulation, Sports performance, Sports training

## Abstract

**Background:**

Soccer coaches often employ small-sided games (SSGs) to elicit both physiological and technical responses from players. However, numerous contextual factors can influence the outcomes of these games. This comparative study aimed to investigate how environmental temperature (< 21ºC and > 29ºC) impacts heart rate responses, perception of effort, and technical performance in young male football players during SSGs.

**Methods:**

This study compares temperatures below 21ºC (∼ 20.4 ± 0.4ºC) with temperatures above 29ºC (∼ 29.7 ± 0.6ºC). This repeated measures study design involved 60 male football players at a trained/developmental level, selected from under-16 and under-19 teams. It aimed to assess the effects of the 3v3 format, conducted repeatedly under conditions of 21ºC and above 29ºC. Throughout the games, mean heart rate responses (HRmean), measured via heart rate sensors; rate of perceived exertion (RPE), assessed using the CR-10 Borg scale; and successful passes and lost balls, tracked through an ad hoc observational analysis tool, were monitored.

**Results:**

No significant interactions were observed (time*age group) in meanHR (*F* = 0.159; *p* = 0.691; $\:{\eta\:}_{p}^{2}$=0.003), RPE (*F*=0.646; *p*=0.425; $\:{\eta\:}_{p}^{2}$=0.011), number of passes completed (*F*=0.204; *p*=0.654; $\:{\eta\:}_{p}^{2}$=0.003), and number of lost balls (*F* = 0.157; *p* = 0.694; $\:{\eta\:}_{p}^{2}$=0.003). Overall, significantly higher heart rate responses in mean HR (*p*<0.001) and RPE (*p*<0.001) were observed at temperatures above 29ºC, while significantly more passes were completed at temperatures below 21ºC (*p*<0.001).

**Conclusions:**

Heat conditions significantly intensified the psychophysiological responses in players, concurrently leading to a significant impairment in the number of passes. Coaches should contemplate implementing mitigation strategies to avert performance declines during heat conditions when utilizing SSGs.

## Introduction

Football player performance is inherently influenced by various factors that interact to shape their performance on the field [1]. Among these factors, the dynamics of a match, situational variables, and environmental conditions play crucial roles in determining players’ responses during gameplay [2]. Temperature emerges as a significant environmental determinant, with extreme conditions like cold and hot environments having noticeable effects on performance during matches [3, 4].

In hot temperature environments, several aspects of football performance are impacted. Physiologically, players face increased thermoregulatory demands, leading to elevated heart rates, dehydration, and reduced cardiovascular efficiency [5]. These conditions can exacerbate fatigue and decrease endurance, ultimately affecting players’ overall output during matches [6]. Additionally, physical attributes such as sprinting speed [7] and jumping ability [8] may decline due to heat-induced muscle fatigue and diminished energy levels. Furthermore, high temperatures can impair cognitive function, decision-making, and coordination, thereby influencing technical aspects such as passing accuracy and ball control [9].

Studies examining football match performance have indicated a notable decline in player effectiveness when heat temperatures [1, 10]. Research suggests a marked increase in low-intensity activities and prolonged exercise sessions below 85% of maximum heart rate during high temperatures (> 29ºC) compared to more moderate conditions [4]. Also, adverse effects of hot weather on key performance metrics such as high-speed running and sprinting have been observed [11]. Regarding technical performance, the findings are somewhat conflicting. While certain studies failed to identify significant differences in the effectiveness of technical actions under varying thermal conditions [12], others highlighted impairments such as an increase in lost ball occasions [13].

While the evidence regarding heat temperature’s impact on football matches is well-established, as summarized in two dedicated systematic reviews [10, 14], original research on training contexts lacks robustness. Specifically, when considering the implementation of game-based training drills such as small-sided games (SSGs) [15–17], it is important to gain insights into how environmental conditions can interact with task constraints to influence the ultimate response of players. SSGs are frequently employed by soccer coaches as a multifaceted training drill with various objectives [18]. They can provide a high-intensity workout to elicit an appropriate aerobic power stimulus [19] while also facilitating players’ frequent practice of specific technical skills [20] and execution of specialized tactical behaviors [21].

SSGs can be categorized as drill-based or ecological-based games [22], wherein the coach adjusts task conditions to highlight specific behavioral changes in players, ultimately affecting their physical and physiological exertion levels during gameplay. The most common conditions utilized by coaches include the format of play and pitch size, which frequently result in varying physical and physiological responses, as extensively documented in the literature [17]. For instance, smaller formats ranging from 1v1 to 4v4 typically induce significantly higher heart rates and perceived exertion levels, often exceeding 85% of maximal heart rate [23]. Moreover, modifying pitch sizes can have a notable impact on the physical engagement in these games, as larger relative areas per player (> 100 m^2^) are typically associated with significantly greater total distances covered, as well as increased distances covered at higher speed intensities [24, 25]. Additionally, smaller SSGs tend to promote a higher number of technical actions (e.g., passes) compared to larger formats [26]. By addressing tactical, technical, physical, and physiological demands in a holistic manner, these games are widely integrated into soccer training sessions, frequently employed (2–3 sessions a week, 16–30 min/session) across both youth and adult training programs [27].

Despite the extensive reporting on SSGs, research focusing on environmental conditions that can ultimately influence performance in these games is scarce. One of the few studies [28] examined the effects of pre-cooling on ten professional football players exposed to temperatures of 30 ± 2ºC and 75 ± 5% relative humidity. It was observed that the physiological and perceptual benefits of pre-cooling in the field were minimal [28]. However, this study [28] did not specifically address the effects of heat conditions on players’ performance during SSGs.

Monitoring the impact of heat on heart rate responses using heart rate monitors is crucial for understanding the physiological mechanisms of thermoregulation and cardiovascular strain, which can negatively affect exercise performance [29]. This knowledge helps in developing strategies to optimize performance and prevent heat-related issues. Additionally, analyzing the rate of perceived exertion (RPE) is also important, as it can shed light on how heat stress influences perceived effort and aid in creating interventions to manage exertion and performance under such conditions [30]. Finally, given the importance of technical performance in these games, especially since technical and tactical responses are key objectives of SSG, understanding the effects of heat on these parameters provides valuable insights for coaches [31].

Given that SSGs are determined by the synergistic interaction between task conditions and their interplay with player characteristics and environmental factors [32], and considering that most training sessions are not specifically regulated to the same extent as matches, it becomes imperative to expand original research efforts to understand how training drills may be influenced by environmental temperatures, particularly hot conditions which could impair drill performance and ultimately affect training adaptations. Recognizing the significance of this topic and aiming to offer valuable insights for coaches and sports scientists, this study aims to compare the effects of hot versus moderate environmental temperatures on male soccer players’ heart rate responses, perceived effort, and technical performance. It is hypothesized that heart rate responses and perceived exertion will be significantly higher during hot conditions [4], while lost balls [13] will also increase in hot conditions.

## Methods

This study adhered to the Strengthening the reporting of observational studies in epidemiology (STROBE) guidelines [33] for reporting cross-sectional study designs.

### Ethics

Both the participants and their legal guardians were briefed on the study protocol, associated risks, and potential benefits. Their legal guardians signed an informed consent for participation, which explicitly stated their right to withdraw from the study at any time without penalty. The study received approval from the Chengdu Sport University under code number 134/2023 and adhered to the ethical standards outlined in the Declaration of Helsinki regarding research involving human subjects.

### Study design

**Fig. 1:**
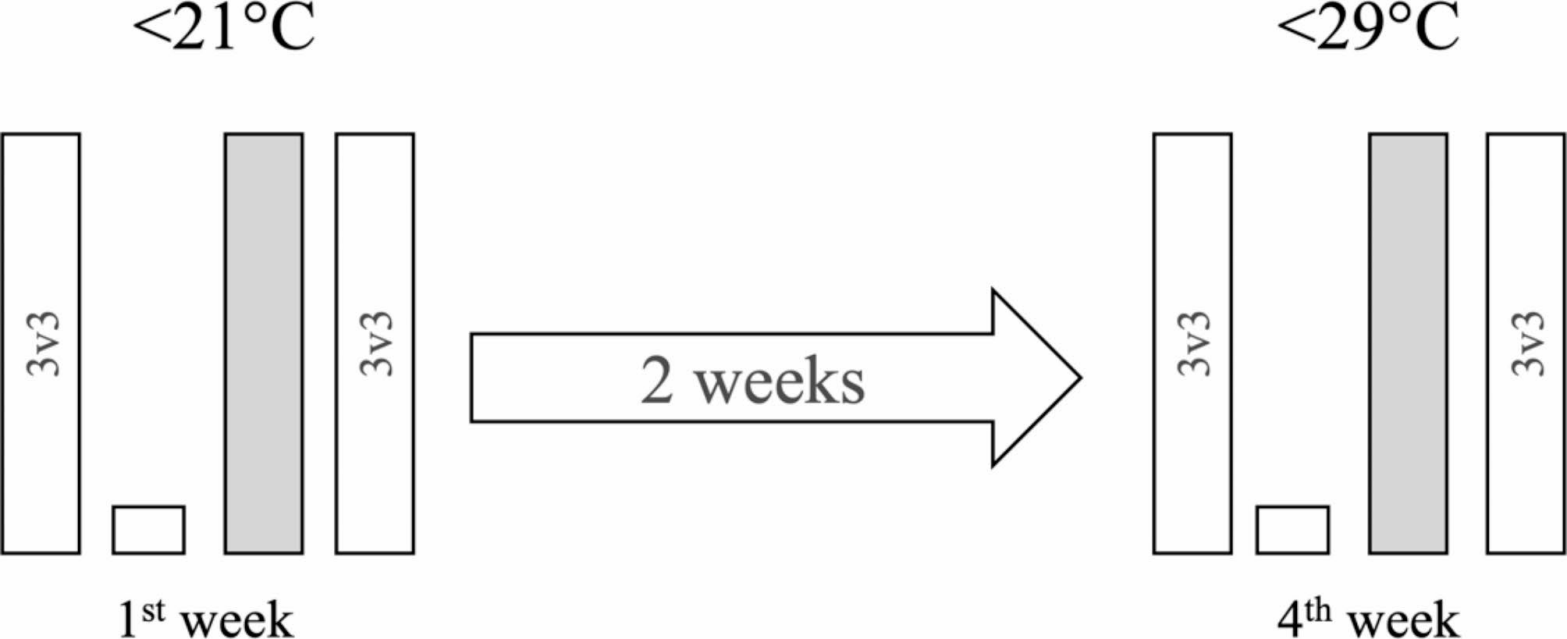
Repeated measures design. <21ºC: Conditions where the temperature during small-sided games was below 21ºC; >29ºC: Conditions where the temperature during small-sided games was above 29ºC. 3v3: format of play of 3v3

This study employed a repeated measures design (Fig. 1), wherein the same players engaged in the same small-sided game (SSG) format (3v3) under two distinct conditions: (i) outdoor wet bulb globe temperature below 21ºC, and (ii) outdoor wet bulb globe temperature above 21ºC. Under each condition, the players took part in two training sessions in the same week (interspaced by two day) during which they completed two repetitions of the SSG format.

### Setting

The study was conducted during the second week of June and the first week of July in Chengdu. The location was chosen for convenience, as the anticipated temperatures aligned with the study’s protocol and the teams were available to participate. There was a difference of two weeks between the sessions conducted on < 21ºC, and those > 29ºC, although the data collection in both conditions occurred in the same days of the week. In summary, there was a 14-day interval between session 1, which was conducted in < 21ºC using the 3v3 format, and session 3, which took place in temperatures exceeding 29ºC. Similarly, there was also a 14-day gap between session 2, held in < 21ºC using the 3v3 format, and session 4, conducted in temperatures exceeding 29ºC.

### Participants

Following a convenience sampling method, this study recruited 60 players from four teams of male youth soccer players aged under-16 (U16) and under-19 (U19), competing at a trained/developmental level within a regional context (Chengdu). The participants were selected based on the following inclusion criteria: (i) being male, to ensure more homogeneous biological responses and to align with the study’s objectives; (ii) being youth between 16 and 19 years old at the moment of the start of the study, an age range crucial for specialization in the training process; (iii) being outfield players; (iv) participating in all data collection moments related to the study; (v) abstaining from drugs or stimulating drinks throughout the study period; and (vi) maintaining good health without any injuries or illnesses during the study period was essential to prevent any health-related factors from biasing the response to the conditions being studied.

Participants were excluded from the study if they did not meet specific criteria: (i) females were excluded to ensure homogeneous biological responses; (ii) goalkeepers; (iii) participants who missed any of the scheduled data collection sessions were excluded to maintain the study’s integrity; (iv) individuals who used drugs or stimulating drinks during the study period were excluded to avoid potential confounding effects; and (v) participants experiencing injuries or illnesses during the study were excluded to ensure that health-related factors did not bias the results.

Participants had similar training backgrounds, typically undergoing training three times a week for 100–110 min each session, in addition to matches.

### Variables

The temperature classification (i.e., < 21ºC and > 29ºC) was considered an independent variable. The temperature was assessed on-site using a handheld wet bulb globe thermometer featuring a 4 cm globe (model WBGT8758, General Tools & Instruments Co.), which was calibrated before each data collection session in accordance with the manufacturer’s recommendations. This device gauges ambient temperature, relative humidity, radiant heat, and wind speed. The research was conducted in Chengdu.

Prior to conducting the study, it was determined that the investigation would focus on conditions both below 21ºC and above 29ºC, with the objective of examining how temperature variations may impact performance, referencing the threshold of 29ºC established in previous study [4]. While it was not possible to precisely select the conditions, the research team waited for the most favorable conditions to meet the eligibility criteria.

Under conditions where the temperature was below 21ºC, the first session recorded a temperature of 20.7ºC (relative humidity of 65.8%), while the second session registered 20.1ºC, resulting in an average of 20.4 ± 0.4ºC (relative humidity of 62.3%). Conversely, when the temperature exceeded 29ºC, the first session measured 29.2ºC (relative humidity of 59.3%), and the second session recorded 30.1ºC, yielding an average of 29.7 ± 0.6ºC (relative humidity of 57.3%).

### The small-sided game

The 3v3 format was implemented on a 26 × 22 m field (95.3 m^2^/player). The objective of the game was to score in a small target measuring 2 × 1 m. No offside rule was enforced, and players repositioned the ball using their feet. All other rules remained consistent with those of the official game. Each team comprised one defender, one midfielder, and one attacker whenever possible. The composition of teams remained unchanged throughout the data collection sessions, and they competed against the same opponents to ensure the reproducibility of conditions. The small-sided game format was utilized twice, each lasting 3 min with a 3-minute rest period, following a standardized warm-up protocol. The warm-up protocol consisted of 8 min of running (at moderate intensity, between 60 and 65% of maximal heart rate), followed by 6 min of dynamic stretching. The dynamic stretching routine included two exercises focusing on hamstrings, two on quadriceps, one targeting adductors, one for abductors, and one for the gastrocnemius. Additionally, participants performed 3 min of short 5-meter accelerations, decelerations, and reactive jumps (10 repetitions of pogo jumps). Before the end of the warm-up and the start of the 3v3 format, a 3-minute rest period was allowed.

The SSGs took place on synthetic turf, consistently in the same location (i.e., the same pitch) and time (5 p.m.). No coaching encouragement was provided during the sessions. The coach’s involvement was limited to assigning players to teams, with the objective of perceptively organizing teams in a more competitive manner. These decisions were primarily guided by the players’ skill levels.

### Outcome measures

Throughout all the 3v3 games, players were continuously monitored using individual heart rate sensors, recording data at 1-second intervals (H10 Bluetooth, Polar Electro Oy, Kempele, Finland). The Polar H10 heart rate monitor demonstrates strong agreement with other heart rate systems, making it highly recommended for accurate heart rate measurements [34]. Mean heart rate was calculated over the duration of the games and used for further data analysis.

Additionally, immediately following the conclusion of each game, the CR10 Borg scale [35], translated into the players’ native language, was utilized to gauge each player’s perceived effort during the game. The scale’s validity and reliability for assessing exercise intensity have been confirmed [36]. This scale was administered individually, with players responding to the question, “How intense was the exercise?” Their responses (score) were recorded in a specially designed form. The form was developed by creating an image that combines numerical values with verbal anchors. Subsequently, the player is presented with a box where they are required to input the score corresponding to the exercise in response to the question about its intensity. Players were already familiar with the scale as it was a routine part of their training. The CR10 Borg scale scores from each game were utilized for subsequent data analysis.

An ad hoc observational tool, externally validated by two experts, was developed specifically to track and record the number of passes completed by each player, as well as the number of balls lost. A completed pass was counted whenever a player successfully delivered the ball to a teammate, who then received correctly maintaining their possession up to perform a next action. Conversely, a lost ball was considered whenever a player either lost control of the ball themselves, was dispossessed by an opponent, or failed to complete a pass.

The observations were conducted by a single observer with over 3 years of experience in sports training and a degree in sports sciences. Additionally, the observer’s intra-rater reliability was assessed through an exploratory study involving data from two games. The same games were analyzed on two separate occasions, spaced 25 days apart. The intraclass correlation coefficient test revealed values exceeding 0.90, confirming the reliability of the evaluator.

The games were recorded using a Xiaomi Mi Action Camera (HD 4 K 2160p, 30 fps; Xiaomi, China), strategically positioned to capture all game actions without the need for camera movement. The number of passes completed and lost balls per player were ecorded and registered for further data analysis.

### Study size

The a priori sample size was calculated using G*Power (version 3.1.9.6, University of Düsseldorf, Germany) [37] with a partial effect size of 0.420, as reported in a previous study [4] comparing the effects of heat conditions on physiological responses in soccer. This calculation determined an effect size 𝑓 of 1.4. Combining this effect size with a power of 0.95, for 2 groups and 2 measurements, resulted in a recommended total sample size of 6. With the current sample of 60, it was achieved a power of greater than 0.999 in repeated measures.

### Statistical analysis

The average values obtained for each outcome in each temperature condition (< 21ºC and > 29ºC) were used as measurements for the inferential statistics. Within-player variability was calculated by employing the coefficient of variation, expressed as a percentage for each specific condition, integrating the individual results of all sessions and repetitions conducted. Moreover, descriptive statistics are presented as means and standard deviations. The normality of the sample was assessed and confirmed using the Kolmogorov-Smirnov test (*p* > 0.05), while homogeneity was verified through Levene’s test (*p* > 0.05). A mixed ANOVA (time*group) was employed to compare the performance of the same players under two different conditions (< 21ºC and > 29ºC). The average performance of players in games conducted under each condition served as the data for analysis. To gauge the magnitude of the effect size, the partial effect size ($\:{\eta\:}_{p}^{2}$) was calculated [38]. Cohen’s d effect size was used to calculate the magnitude of differences in pairwise comparisons. The magnitudes (d) were interpreted as follows [38]: 0.0-0.02, trivial; 0.2–0.5, small; 0.5–0.8, medium; and ≥ 0.8, large.

All statistical analyses were conducted using SPSS software (IBM SPSS Statistics for Windows, Version 28.0., IBM Corp., Armonk, New York, USA), with significance set at *p* < 0.05. The visualization illustration was generated using JASP software (version 0.18.3, University of Amsterdam, The Netherlands).

## Results

Table 1 shows the key demographic information of the participants. On average, the U16 players (*N* = 30) were 16.3 ± 0.3 years old, with an average of 5.1 ± 0.9 years of soccer experience, whereas the U19 players (*N* = 30) were 17.6 ± 0.3 years old, with an average of 6.8 ± 1.2 years of soccer experience.

**Table 1:** Main demographic characteristics of the participants

	Under-16	Under-19	Overall
Sample size (N)	30	30	60
Age (years)	16.3 ± 0.3	17.6 ± 0.3	17.0 ± 0.7
Body mass (kg)	62.1 ± 6.7	64.4 ± 7.1	63.3 ± 7.0
Height (cm)	175.2 ± 4.6	176.5 ± 5.3	175.9 ± 5.0
Soccer experience (years)	5.1 ± 0.9	6.8 ± 1.2	6.0 ± 1.4

In the condition below 21 °C, the mean coefficient of variation of the mean heart rate within players was 1.3% ± 0.3%, whereas in the condition above 29 °C, it was 1.1% ± 0.5%. Similarly, in the below 21 °C condition, the mean coefficient of variation of the CR10 Borg scale within players was 4.1% ± 1.5%, while in the above 29 °C condition, it was 5.0% ± 2.0%. In the condition below 21 °C, the mean coefficient of variation of the number of passes within players was 26.5 ± 10.2%, whereas in the condition above 29 °C, it was 32.9 ± 12.4%. Similarly, in the below 21 °C condition, the mean coefficient of variation of the balls lost within players was 69.0 ± 39.5%, while in the above 29 °C condition, it was 78.5 ± 26.0%.

No significant interactions were observed (time*age group) in meanHR (*F* = 0.159; *p* = 0.691; $\:{\eta\:}_{p}^{2}$=0.003), RPE (*F*=0.646; *p*=0.425; $\:{\eta\:}_{p}^{2}$=0.011), number of passes completed (*F*=0.204; *p*=0.654; $\:{\eta\:}_{p}^{2}$=0.003), and number of lost balls (*F* = 0.157; *p* = 0.694; $\:{\eta\:}_{p}^{2}$=0.003).

**Fig. 2:**
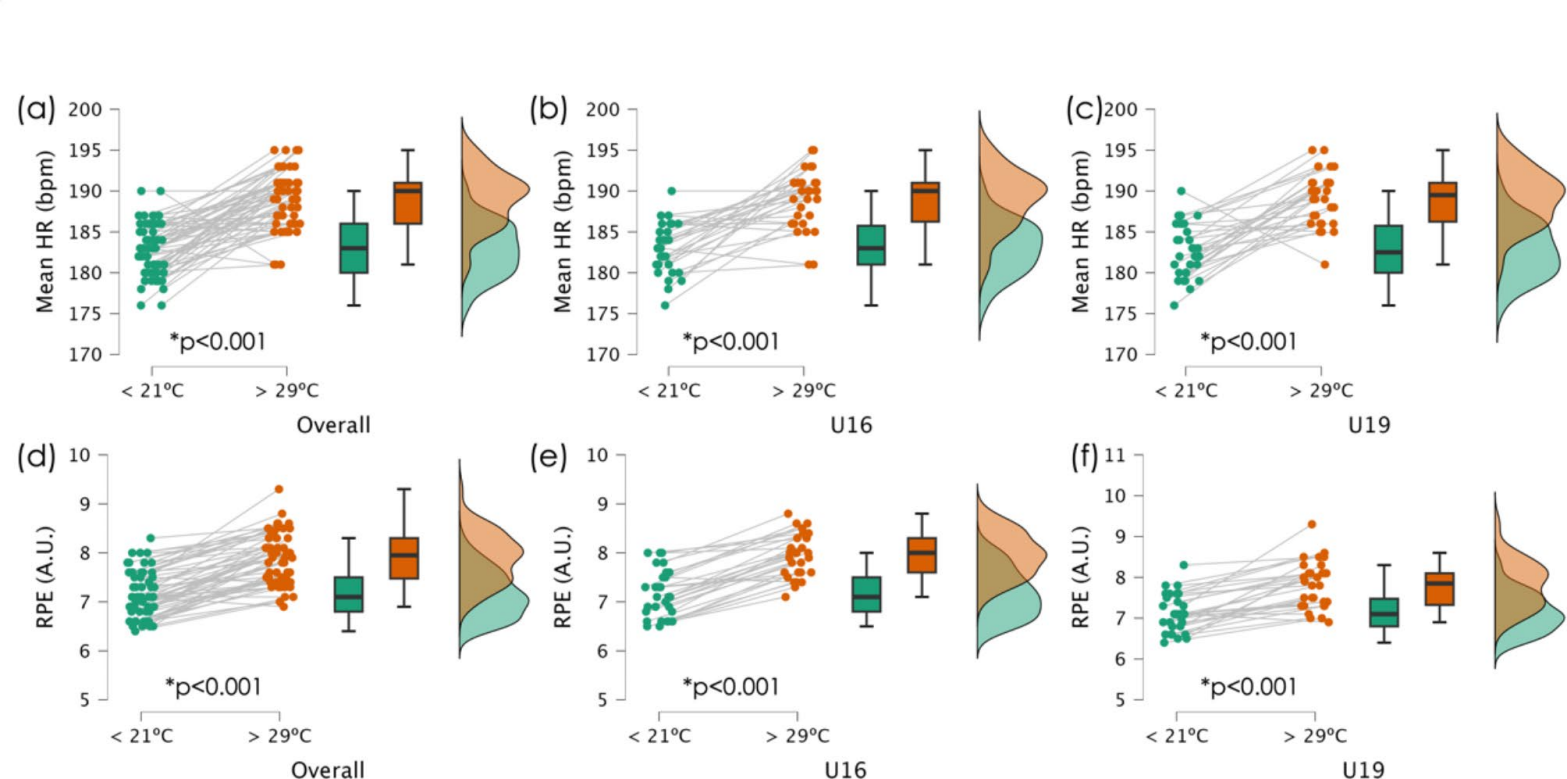
Descriptive statistics of the mean heart rate (HR) and rate of perceived exertion (RPE) for the overall participants, and split by age group (under-16, U16; and under-19, U19). *Significantly different within-group. (**a**) meanHR of the overall group; (**b**) meanHR of the under-16; (**c**) meanHR of the under-18; (**d**) RPE of the overall group; (**e**) RPE of the under-16; (**f**) RPE of the under-19

**Fig. 3:**
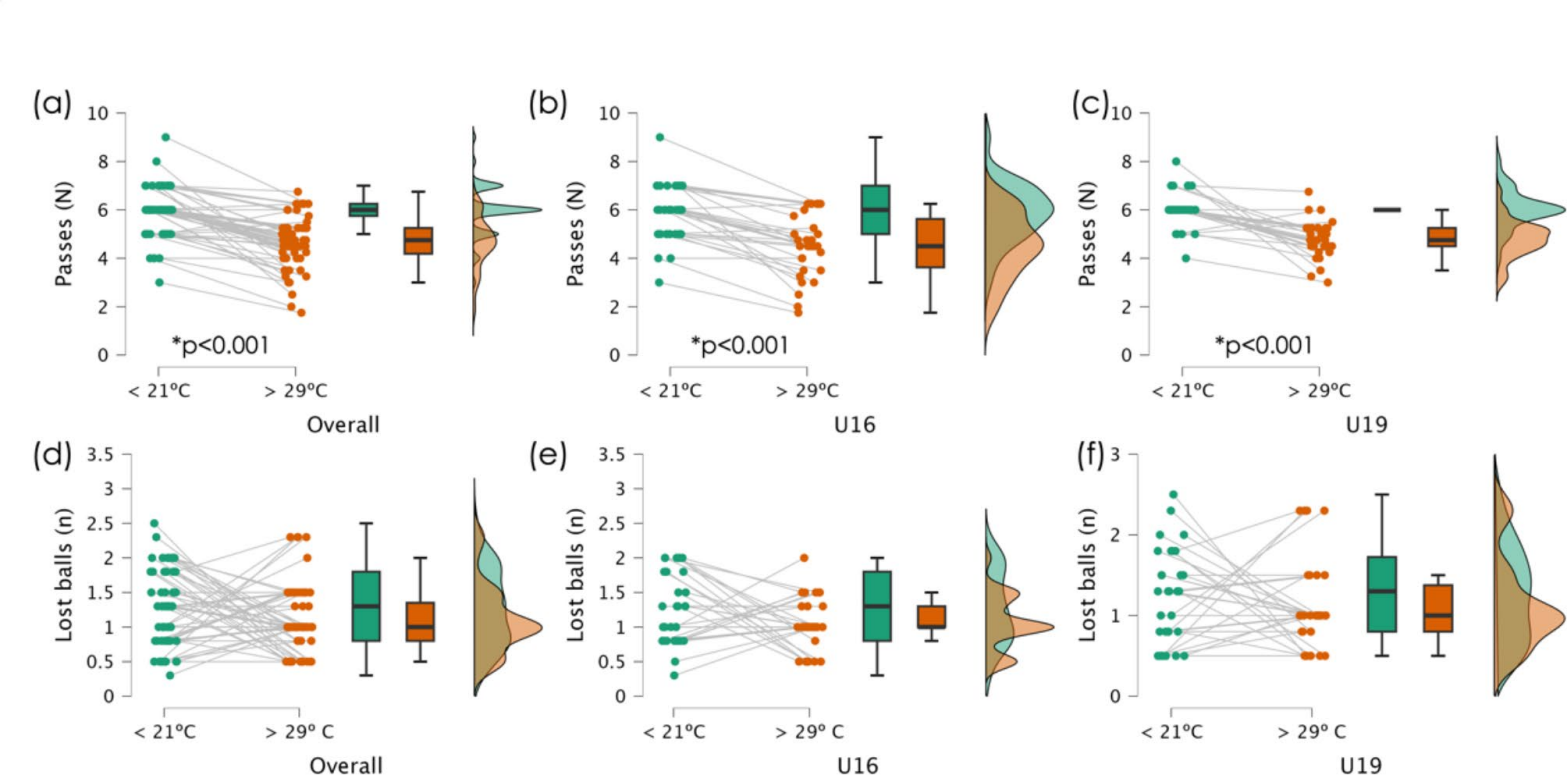
Descriptive statistics of the passes completed and lost balls for the overall participants, and split by age group (under-16, U16; and under-19, U19). *Significantly different within-group. (**a**) passes completed of the overall group; (**b**) passes completed of the under-16; (**c**) passes completed of the under-18; (**d**) lost balls of the overall group; (**e**) lost balls of the under-16; (**f**) lost balls of the under-19

No significant differences were found between age groups at < 21ºC for the meanHR (*F* = 0.160; *p* = 0.690; $\:{\eta\:}_{p}^{2}$=0.003), RPE (*F*=0.108; *p*=0.743; $\:{\eta\:}_{p}^{2}$=0.002), number of passes completed (*F*=0.268; *p*=0.607; $\:{\eta\:}_{p}^{2}$=0.005), and number of lost balls (*F* = 0.020; *p* = 0.888; $\:{\eta\:}_{p}^{2}$<0.001). Moreover, no significant differences were found between age groups in conditions > 29ºC in the meanHR (*F* = 0.036; *p* = 0.851; $\:{\eta\:}_{p}^{2}$=0.001), RPE (*F*=1.015; *p*=0.318; $\:{\eta\:}_{p}^{2}$=0.017), number of passes completed (*F* = 0.851; *p* = 0.360; $\:{\eta\:}_{p}^{2}$=0.014), and number of lost balls (*F* = 0.265; *p* = 0.609; $\:{\eta\:}_{p}^{2}$=0.005). Figure 2 shows the statistics for the meanHR and RPE across all participants, while Fig. 3 shows the number of passes completed and balls lost.

Overall, significantly higher heart rate responses in mean HR (*p* < 0.001) and RPE (*p* < 0.001) were observed at temperatures above 29ºC, while significantly more passes were completed at temperatures below 21ºC (*p* < 0.001). The descriptive statistics for all players, as well as those organized by age groups, are shown in Table 2.

**Table 2:** Descriptive statistics and comparisons of the outcomes analyzed considering the two temperature conditions

	Temperature < 21ºC	Temperature > 29ºC	Mean difference | *p*-value | d
Under 16			
Mean heart rate (bpm)	183.0 ± 3.1	189.0 ± 3.5	6.9 | *p* < 0.001 | d = 1.82
CR10 Borg (A.U.)	7.2 ± 0.5	8.0 ± 0.4	0.8 | *p* < 0.001 | d = 1.78
Passes (n)	5.9 ± 1.2	4.5 ± 1.3	1.4 | *p* < 0.001 | d = 1.12
Lost balls (n)	1.3 ± 0.5	1.1 ± 0.4	0.2 | *p* = 0.157 | d = 0.44
Under 19			
Mean heart rate (bpm)	182.7 ± 3.3	189.1 ± 3.3	6.5 | *p* < 0.001 | d = 1.83
CR10 Borg (A.U.)	7.1 ± 0.5	7.8 ± 0.6	0.7 | *p* < 0.001 | d = 1.56
Passes (n)	6.0 ± 0.8	4.8 ± 0.8	1.2 | *p* < 0.001 | d = 1.50
Lost balls (n)	1.3 ± 0.6	1.1 ± 0.6	0.1 | *p* = 0.386 | | d = 0.33
Overall			
Mean heart rate (bpm)	182.8 ± 3.2	189.1 ± 3.4	6.2 | *p* < 0.001 | d = 1.83
CR10 Borg (A.U.)	7.1 ± 0.5	7.9 ± 0.5	0.7 | *p* < 0.001 | d = 1.78
Passes (n)	6.0 ± 1.0	4.7 ± 1.1	1.3 | *p* < 0.001 | d = 1.04
Lost balls (n)	1.3 ± 0.5	1.1 ± 0.5	0.1 | *p* = 0.108 | d = 0.44

bpm: beats per minute; A.U.: arbitrary units; n: number

## Discussion

The study aimed to compare the effects of high and moderate environmental temperatures on heart rate responses, perceived effort, and technical performance in male soccer players. The findings show that higher temperatures (above 29ºC) significantly increase heart rate responses and perceived exertion, and lead to a notable reduction in completed passes. However, the frequency of lost balls did not differ significantly between temperature conditions. These results indicate that elevated temperatures impair technical performance while increasing physiological stress in soccer players.

The study showed that heat conditions significantly raised heart rate responses during SSGs. Although these results are not directly comparable to previous SSG studies, they are consistent with similar evidence observed in match conditions [39], although the evidence is contradictory [9]. In heat conditions exceeding 29 °C, the significant increase in heart rate responses during 3v3 compared to regular conditions at 21 °C can be attributed to several physiological and thermoregulatory mechanisms. Elevated environmental temperatures induce greater heat accumulation within the body during exercise, leading to heightened core temperature [40]. Consequently, to dissipate this excess heat, the body initiates vasodilation of peripheral blood vessels, directing more blood flow to the skin for convective heat loss through sweating [41]. However, in some conditions as limited airflow or high relative humidity, evaporative cooling becomes less efficient, resulting in a compromised ability to regulate core temperature [42]. As a compensatory response, the cardiovascular system intensifies its efforts to maintain thermal equilibrium by increasing cardiac output [43]. This involves elevating heart rate to facilitate greater blood circulation, enhancing convective heat transfer to the skin surface for dissipation into the environment [44]. Moreover, the metabolic demands of exercise during 3v3 exacerbate heat production, amplifying the strain on the cardiovascular system due to being a highly metabolic drill [19].

Similarly with the increases in heart rate, rate of perceived exertion was also significantly higher in heat conditions. This study aligns with previous research reports which described significantly greater perceived loads in training sessions which has heat conditions [45]. The significantly greater rate of perceived exertion observed in heat conditions can be explained through various interconnected psychophysiological mechanisms. Primarily, the elevation in environmental temperature exacerbates physiological strain during exercise, prompting intensified sensations of effort and discomfort [46]. Furthermore, the central governor model posits that the brain acts as a regulator of exercise performance, integrating sensory feedback to modulate effort perception and preserve homeostasis [47]. In heat conditions, afferent signals from thermoreceptors, alongside elevated heart rate and respiratory responses, converge to amplify the perception of effort as the brain interprets these signals as indicators of physiological stress and imminent fatigue [48]. Moreover, the psychological component of perceived exertion is influenced by environmental factors, such as thermal discomfort and perceived environmental threat [43], which can further augment rate of perceived exertion in heat conditions.

In addition to the psychophysiological responses investigated in the current study, significant decreases in the number of completed passes were observed during SSGs conducted in hot conditions, along with an increase in the number of unsuccessful passes. The effects of heat conditions on technical performance are conflicting, with some studies not revealing a meaningful impact [12], while others demonstrate increases in instances of lost possession [13]. The significant reduction in the number of passes and the increase in unsuccessful ones observed in the current study can have some possible explanations. Elevated environmental temperatures impose physiological stressors that can impair cognitive function, including decision-making abilities and technical performance [49]. Research suggests that heat stress compromises cognitive function by impeding neural processing speed and accuracy, thereby impeding the ability to perceive and execute decisions effectively [50]. This impairment stems from alterations in synaptic transmission efficiency and neurotransmitter release, resulting in delayed and erratic neural responses [51]. Consequently, athletes experience difficulty in perceiving situational cues and formulating appropriate responses, leading to suboptimal decision-making on the field [49].

Additionally, heat-induced dehydration, even at modest levels, has been shown to detrimentally affect cognitive function, further exacerbating errors in decision-making and technical execution [52]. Dehydration may disrupt the balance of electrolytes and fluid volume essential for optimal neural functioning. As body water levels decline, electrolyte concentrations become disproportionately elevated, disrupting the electrochemical gradients necessary for efficient neuronal transmission [53]. This disruption may impair synaptic firing rates, slows neural conduction velocity, and compromises the accuracy and speed of cognitive processing [54]. Moreover, dehydration-induced alterations in cerebral blood flow dynamics may exacerbate cognitive deficits by compromising cerebral perfusion and metabolic activity [48].

Moreover, the increased cardiovascular strain associated with heat stress may divert cognitive resources away from strategic decision-making towards basic physiological processes, limiting the cognitive bandwidth available for complex motor tasks such as passing [49]. Furthermore, the perception of effort and discomfort induced by heat stress can influence athletes’ motivation and willingness to engage in high-intensity technical maneuvers, leading to a more conservative playing style characterized by fewer passes and simpler plays [55]. This may also account for the significant decrease in the number of shots on goal during hot conditions.

Despite the innovative nature of this study, several limitations should be acknowledged. One significant limitation is the focus exclusively on male youth categories, which may limit the generalizability of the findings to other populations, such as elite adult athletes or females. Additionally, the study was restricted to examining only ambient temperature at the start of the match, without considering other potentially influential atmospheric conditions such as atmospheric pressure, wind, humidity, and precipitation. These factors, along with the potential for temperature fluctuations during the game, particularly if the kick-off time varies, might also impact physical performance.

Moreover, the analysis did not account for external load demands, which could offer further insights into the physiological changes observed. The heterogeneity introduced by players spanning different age groups and the lack of exploration into their cardiorespiratory fitness levels might affect individual responses to environmental stressors like heat. Future research should incorporate an analysis of physical fitness as a potential moderator of heat stress effects during SSGs. Additionally, situational variables such as game location, result status, and team quality, which could modulate the impact of environmental conditions, were not considered in this study [56]. To provide a more comprehensive understanding, future studies should examine these variables and their interactions with heat stress. Extending the analysis to different types of SSGs and exploring their acute effects in varying heat conditions would also be beneficial. Investigating physiological mechanisms and thermoregulatory processes, including hydration status and fluid losses relative to performance, could offer deeper insights into the observed effects. Addressing these aspects will enhance the understanding of how environmental conditions impact performance and provide more actionable recommendations for managing heat stress in SSGs.

Despite these limitations, this research contributes to the understanding of how elevated temperatures impact both the physiological and technical aspects of soccer performance during SSG training drills. It was found that temperatures exceeding 29ºC significantly elevate heart rate responses and perceived exertion during SSGs, while also reducing the number of successful passes completed. This highlights a critical interaction between environmental heat and player’s performance, emphasizing that high temperatures impose a substantial physiological stress on players. Theoretically, these findings align with existing literature on heat stress, reinforcing the notion that elevated temperatures exacerbate cardiovascular strain and perceived effort through mechanisms like increased heart rate and impaired thermoregulation. Additionally, this study extends the current body of knowledge by revealing that while heat does not significantly affect ball loss frequency, it does impair technical performance, particularly in terms of passing accuracy. Practically, these results highlight the need for strategic adaptations in training strategies under heat stress conditions. Coaches and sports scientists should consider these heat-induced changes in physiological and cognitive performance when designing training regimens and game plans, potentially incorporating cooling strategies and hydration interventions to mitigate the adverse effects of heat on soccer performance.

## Conclusions

This study revealed that heat conditions (> 29 °C), compared to moderate temperatures (< 21 °C), significantly intensified both heart rate responses and the perceived exertion reported by players. Additionally, the number of passes was significantly affected by the heat condition, although the number of lost balls did not show a significant difference between temperature conditions. Coaches should consider the need to mitigate heat conditions during training sessions when incorporating SSGs, specifically by implementing pre-cooling techniques and ensuring adequate hydration levels among players.

## Data Availability

The data is available upon reasonable request to the corresponding author.

## Abbreviations

SSGs: Small-Sided Games
